# A dataset that integrates Beach Litter, Beach Uses, and Coastal Oceanographic variables

**DOI:** 10.1038/s41597-025-05321-0

**Published:** 2025-06-11

**Authors:** Bruna De Ramos, Monica Ferreira da Costa

**Affiliations:** 1 Oceanography Departament, Universidade Federal de Pernambuco - UFPE, Recife, Pernambuco Brazil; 2 Coastal Sea Geography Group, Leibniz-Institute for Baltic Sea Research, Seestraße, 15, Rostock, 18119 Germany

**Keywords:** Environmental impact, Sustainability

## Abstract

Marine litter is a multifaceted environmental issue requiring different data types for a better understanding of the problem. This paper presents a new dataset with approximately 6200 data records that compile different data types and sources focused on beach litter. We accessed beach litter data from *in situ* sampling, litter and beach wrack interaction, beach use, accommodation statistic focused on tourism, and meteo-oceanographic variables. A relational database framework was designed to facilitate data access, integration, and future use. Itamaracá Island, northeast Brazil, was used as Area of Interest, and all data was collected in the same time frame for the region, photos from the beach litter sampled items are also available in a data repository. The dataset demonstrated capability to manage diverse data types and sources, and it is an innovative approach for marine litter since it merges data from different sources, types and could give a holistic view about marine litter problem. This study emphasizes the importance of connecting different data sources for the same timeframe in environmental research.

## Background & Summary

Beach litter is a growing concern worldwide due to its negative impacts on the environment, economy, and marine life^1^. Collecting and organizing beach litter data is crucial for understanding its sources and distribution^2^. Integrating meteorological and oceanographic factors such as winds, waves, grain size, beach slope, and precipitation into a unified dataset for the same location and time frame, could enhance data analysis and comprehension of beach and marine litter dynamics^3,4^.

Regarding data collection, meteo-oceanographic data could be retrieved from models (*e.g*., wind direction and intensity) or meteorological stations, and field sampling (*e.g*., grain size). Beach activities and the presence of Marine Protected Areas (MPA) are also key to contextualizing beach litter data^5^, this type of data could be collected by observation and following a given checklist (*e.g*., BeachLog tool^5^, or Coastal Scenery Evaluation techniques^6^). Also, the interaction with marine litter and the biota, such as algae^7^ and protected species^8^ is a topic that is raising attention and concern in the scientific community and coastal managers. This data could be collected in field campaigns, combined or not with beach litter monitoring or cleanup activities.

Tourism has been identified as a potential source of beach litter in many popular tourist destinations^9–11^. However, there remains a gap in the literature regarding datasets that integrate information from both the tourism sector and beach litter. One possible reason for this gap could be the conflict of interest in disclosing data related to tourism activities and accommodation occupancy and revenue, given that tourism is a lucrative and rapidly growing industry^12^

Public datasets provide, most of the time, a fragmented perspective into marine litter topic. For instance, Oracle was used to store data from necropsies and stomach content analyses to study biological interactions with marine litter^13^. Europe has led the way in establishing strong data management practices, exemplified by the pan-European beach litter database. This database supports the EMODnet Chemistry beach format, enabling the integration of datasets from various protocols and reference systems for marine debris monitoring^14–16^. In contrast, countries in the Global South, such as Brazil, still lack comparable frameworks for effective marine litter data management^17^.

If databases could integrate different types of data, it would represent progress in marine litter studies. One challenge is that the timeframes of different data types do not match. In this paper, we made an effort to collect all data (beach litter, beach use, and meteo-oceanographic factors) within the same timeframe and geographic region. As a result, we compiled an unified dataset, enabling a more complete view about beach litter topic. For each type of data, we used a different collect method, but comparable in space – time dimensions.

**Fig. 1:**
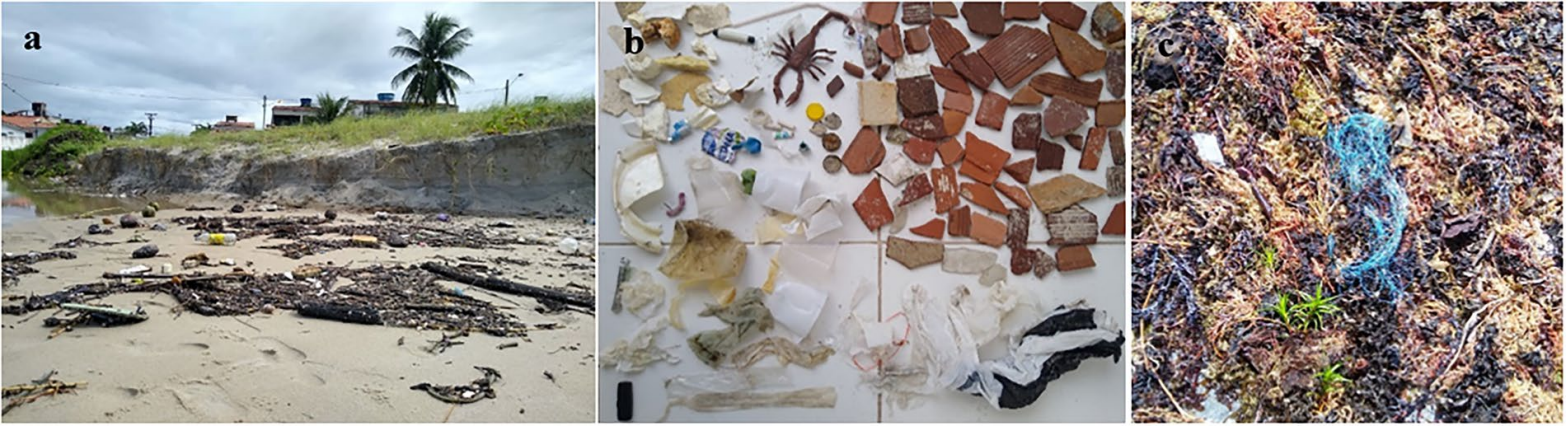
Beach litter in Itamaracá island – Pernambuco – Brazil. (**a**) Beach litter in the sand strip, close to an urban area; (**b**) Beach litter collected in 25 m^2^, (**c**) fishing net fond in a beach wrack in Sossego beach (Sos) in Itamaracá Island.

As a region of interest and for dataset construction, we collected data on Itamaracá Island in northeast Brazil. As shown in Fig. 1(a), beach litter is present along the sand strip and near urban areas, highlighting the importance of integrating different types of data related to beach litter, including beach use. The variety of beach litter possible sources, types and materials (Fig. 1b) also presents a challenge for mitigation and management strategies^18^. Also, the presence of plastic item in beach wrack is reported for the region (Fig. 1c), adding more complexity to management strategies. Connecting data on beach litter, beach use, and coastal oceanographic conditions can provide a more complete understanding of the problem, especially when collected within the same timeframe or sampling effort.

By incorporating variables like meteo-oceanographic data, beach use, and beach litter surveys into a single platform, relational databases enable the identification of correlations and patterns that may not be apparent when analyzing datasets individually. This provides a more comprehensive understanding of marine litter possible sources, society role and impacts, and mitigation strategies. Together with Citizen Science initiatives, it can collaborate for the better use of already collected and open accessed data^19^. Future perspective could include data from initiatives such as ocean travelers (https://serc.si.edu/participatory-science/projects/ocean-travelers) and CoastSnap^20^.

In summary, the main contribution of this paper is that we compiled a dataset for Itamaracá Island integrating different data types focused on beach liter and based on same timeframe data collection.

## Methods

### Area of interest

**Fig. 2:**
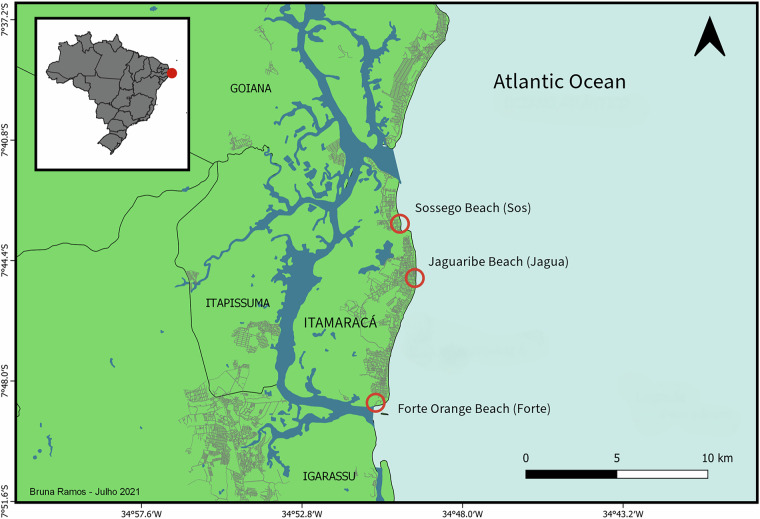
Itamaracá Island and neighbor cities such as Igarassu (south), Itapissuma (west), and Goiana (north). Sampled beaches are represented as red circles and urban areas as gray lines.

Itamaracá Island, situated in the Northeast of Brazil (7.735° S, 34.870° W) (Fig. 2), is a coastal region of significant ecological and socio-economic importance, with a growing tourism sector. The island’s urban development and increasing human activity make it a hot spot for beach litter studies, as these factors contribute to pollution in the coastal environment. Additionally, the island is surrounded by Protected Areas and an important estuarine system^21^. The interaction between urban occupation, tourism, and adjacent conservation areas presents a unique opportunity to understand beach litter dynamics and its interaction with beach use, the tourism sector, and meteo-oceanographic data.

### Relational database

**Fig. 3:**
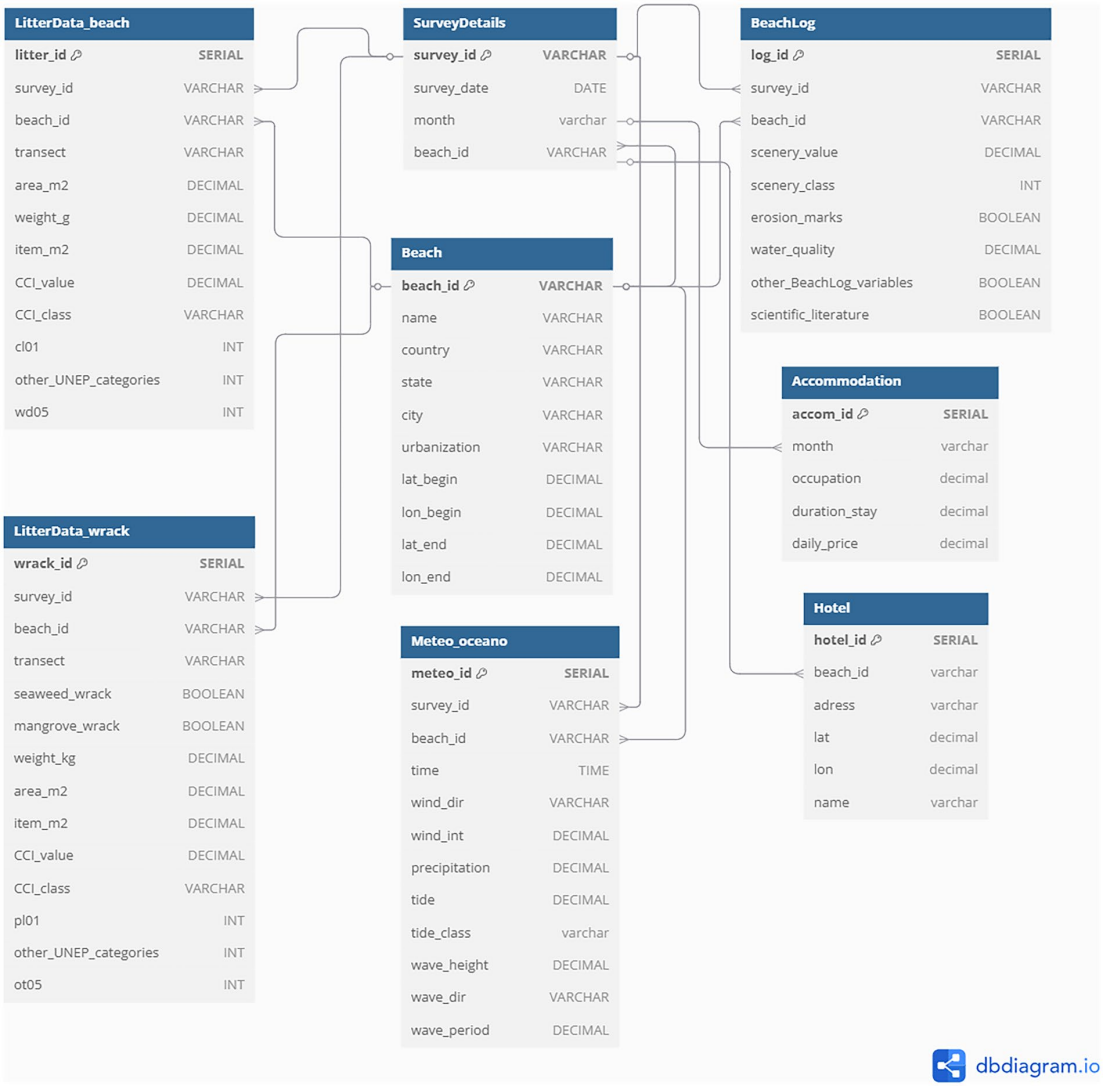
Entity-Relationship Diagram (ERD) for a relational database about beach litter, interaction between litter and beach wrack, accommodation and hotel information, meteo-oceanography, and beach use variables/attributes. Relationships are highlighted in dark blue lines connecting the tables/entities.

We developed a relational database to retrieve and construct a unified dataset focused on beach litter and including other data types. The physical model was implemented in PostgreSQL using the pgAdmin interface and SQL (Structured Query Language). We created the tables, defined their relationships, and input data using a Python script linking the table structure to data organized in a spreadsheet. The database Entity-Relationship Diagram (ERD) is represented in Fig. 3. The Python scripts are available at https://github.com/ramos-bruna/MarineLitter_database.

The data that was used to compile the dataset focused on five aspects: beach litter sampling, litter and beach wrack interaction, beach use, accommodation focused on tourism, and meteo-oceanographic variables (Table 1). All the data was collected on Itamaracá Island in northeast Brazil (Fig. 2). The sampling campaigns occurred during the spring tide of March, June, September, and December 2022.

**Table 1:** Overview of datasets and data sources.

Dataset	Description	Data source
Beach litter	Data on litter quantities. Partially used in a prior study24. (8296 data records)	10.6084/m9.figshare.14128610.v2
	Item per square meter and Clean Coast Index (CCI) (111 records)	New data inserted directly in the dataset LitterData_Beach
Beach Use	BeachLog parameter raw data Collection method based on BeachLog tool^5^ (288 data records)	10.6084/m9.figshare.27246942.v3
	And Coastal Scenery evaluation (24 data records)	New data inserted directly in the dataset BeachLog
Accommodation	Accommodation statistics such as occupation rate, average stay and price. (12 data records)	https://app.airdna.co/data/br/55193?tab=active-str-listings
	Hotel list for each beach (108 data records)	Google Maps Extractor API
Beach litter and beach wrack	Data on presence and absence of seaweed and mangrove wrack, litter items, and the weight of organic material (2849 data records)	10.6084/m9.figshare.28939709.v1
	Clean Coast Index (CCI) (74 data records)	New data inserted directly in the dataset LitterData_wrack
Meteo-oceanographic	Data is inserted directly into the dataset (108 data records)	Tide data recovered in 2022 (https://www.marinha.mil.br/chm/dados-do-segnav/dados-de-mare-mapa) Wave, wind, and precipitation data (GFS data access via NOAA API)

For beach litter, the team walked along a 25-meter transect, collecting all visible litter found between the low tide level and the high tide range. All collected litter was separated, counted, categorized based on UNEP guidelines (2009)^22^ and possible source^23^. Additional details on marine litter collection are described in^24^. The beach litter data used for the compiled dataset is already published at figshare^25^ 10.6084/m9.figshare.14128610.v2 (Table 1) and partially used in a previous study^24^. However, previously only the tables litter_underwater and brand_audit were used. For the compiled dataset presented here we used the table litter_sand and added the Clean Coast Index (CCI) analysis^26^. Additionally, a photo repository from the collected beach litter in December 2022 was created^27^ 10.6084/m9.figshare.28695560.v1.

Beach use data were acquired applying the BeachLog tool^5^ in the beaches in Itamaracá island (Fig. 2) right after the beach litter sampling. The data is available in figshare^28^ 10.6084/m9.figshare.27246942.v2 (Table 1) and it was partially used in a previous publication^5^. However, the BeachLog_itamaraca dataset contain 2 more months of data compared to the previous version of the dataset, and it was used to integrate the compile dataset in the study. Also, we applied the Coastal Scenery evaluation^6^ and added this in the compiled dataset. The Coastal Scenery analysis^6^ was done for the same beaches (Fig. 2) and time frame as the beach litter collection and the BeachLog.

Accommodation focused on tourism data was retrieved from Google Maps and AirDNA (https://www.airdna.co/) for the four months of data collection. The total accommodation options for the island was accounted of 420 units, we subset the hotels in the three sampled beaches for the hotels list. Data as occupation percentage, average stay time and daily price were calculated for the four months of data sampling.

The data collection for the interaction between litter and beach wrack was done on the same date and location (Fig. 2), and the data was retrieved from a dataset in Figshare^29^ 10.6084/m9.figshare.28939709.v1 (Table 1). We applied the CCI analysis for the litter found in the wracks and added this to the compiled dataset.

Regarding meteo-oceanographic variables, tide data were sourced from the Brazilian Navy website (https://www.marinha.mil.br/chm/dados-do-segnav/dados-de-mare-mapa), and the sampling point was Recife Harbor (08° 03′.4 S; 034° 52′.1 W). Wave, wind, and precipitation data were obtained from the Global Forecast System (GSF). The example code for data retrieving is available on https://github.com/ramos-bruna/MarineLitter_database/blob/main/GFS_data_retrieval.py.

## Data Records

All data is provided in a compiled dataset available at figshare^30^ 10.6084/m9.figshare.29128109.v1. The file BeachLitter_compiled_dataset_itamaraca.xlsx contains the sheets Beach, SurveyDetails. LitterData_beach, LitterData_wrack, Meteo_oceano, BeachLog, Accomodation, and Hotel. Additionally, the file data_dictionary_beach_litter_itamaraca.txt describes each sheet from the dataset as well as the columns and respective measurements units.

## Technical Validation

**Fig. 4:**
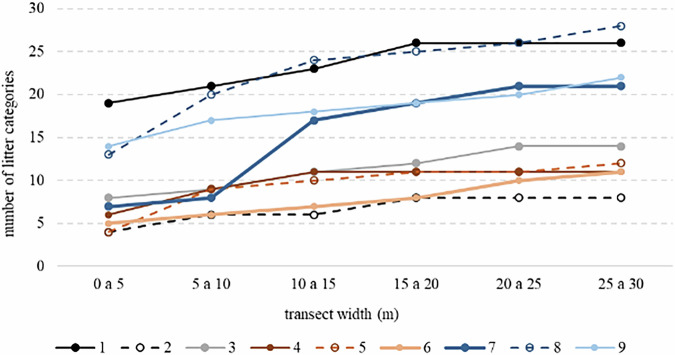
Species accumulation curve applied to beach litter sampled in Itamaracá Island. Each curve represents one sampling effort.

To validate the representativeness of the beach litter sampling effort, the species accumulation curve was applied to beach litter sampling (Fig. 4). This illustrated the relationship between transect width and the number of litter categories recorded. This approach, commonly used in ecology to assess sampling effort^31^, demonstrates that as the sampled area increases, the number of identified litter categories also rises. Each curve represents a different sampling effort, highlighting variations in accumulation patterns across different beaches and transects. From a transect width of 20 m onward, most curves stabilize, with only one remaining unstable at 25 m. This finding validates our data, indicating that more than 90% of the litter types present on the beach were sampled.

The observed differences among curves suggest spatial variability in litter distribution, potentially influenced by factors such as coastal dynamics, human activities, and environmental conditions. These findings highlight the importance of integrating different types of data, such as beach use and meteo-oceanographic variables, to ensure a comprehensive assessment of beach litter composition.

Beach use was validated using an expert’s opinion. Two volunteers with expertise in coastal management tools looked into the data and agreed with the observations collected on the field, based on BeachLog criteria and Coastal Scenery checklist.

## Data Availability

The custom code used in developing the database is accessible at the following repository: https://github.com/ramos-bruna/MarineLitter_database. This includes SQL scripts, data processing scripts in Python, and documentation for the data dictionary.

## References

[1] Wilcox, C., Mallos, N. J., Leonard, G. H., Rodriguez, A. & Hardesty, B. D. Using expert elicitation to estimate the impacts of plastic pollution on marine wildlife. *Mar. Policy* **65**, 107–114 (2016).

[2] Galgani, F. *et al*. Revisiting the strategy for marine litter monitoring within the european marine strategy framework directive (MSFD). *Ocean Coast. Manag.* **255**, 107254 (2024).

[3] Onink, V., Jongedijk, C. E., Hoffman, M. J., Sebille, E. van & Laufkötter, C. Global simulations of marine plastic transport show plastic trapping in coastal zones. *Environ. Res. Lett*. **16** (2021).

[4] Maximenko, N. *et al*. Towards the integrated marine debris observing system. *Front. Mar. Sci*. **6** (2019).

[5] de Ramos, B. & Costa, M. F. D. BeachLog: A multiple uses and interactive beach picture. *Mar. Pollut. Bull.* **193**, 115156 (2023). 37331276 10.1016/j.marpolbul.2023.115156

[6] Ergin, A., Karaesmen, E., Micallef, A. & Williams, A. T. A new methodology for evaluating coastal scenery: Fuzzy logic systems. *Area* **36**, 367–386 (2004).

[7] Robbe, E., Woelfel, J., Balčiūnas, A. & Schernewski, G. An Impact Assessment of Beach Wrack and Litter on Beach Ecosystem Services to Support Coastal Management at the Baltic Sea. *Environ. Manage.* **68**, 835–859 (2021). 34505177 10.1007/s00267-021-01533-3 PMC8578072

[8] Deudero, S. & Alomar, C. Mediterranean marine biodiversity under threat: Reviewing influence of marine litter on species. *Mar. Pollut. Bull.* **98**, 58–68 (2015). 26183308 10.1016/j.marpolbul.2015.07.012

[9] Ali, Q., Yaseen, M. R., Anwar, S., Makhdum, M. S. A. & Khan, M. T. I. The impact of tourism, renewable energy, and economic growth on ecological footprint and natural resources: A panel data analysis. *Resour. Policy* **74**, 102365 (2021).

[10] Cristiano, S. *et al*. Beach landscape management as a sustainable tourism resource in Fernando de Noronha Island (Brazil). *Mar. Pollut. Bull.* **150**, 110621 (2020). 31669708 10.1016/j.marpolbul.2019.110621

[11] Corraini, N. R., Lima, A., de, S., de, Bonetti, J. & Rangel-Buitrago, N. Troubles in the paradise: Litter and its scenic impact on the North Santa Catarina island beaches, Brazil. *Mar. Pollut. Bull.* **131**, 572–579 (2018). 29886984 10.1016/j.marpolbul.2018.04.061

[12] Fosse, J., Kosmas, I. & Gonzales, A. The future of Mediterranean tourism in a (post) covid world. *Eco-Med Brief*. **01** (2021).

[13] Franeker, J. A. V. *et al*. Monitoring plastic ingestion by the northern fulmar Fulmarus glacialis in the North Sea. *Environ. Pollut.* **159**, 2609–2615 (2011). 21737191 10.1016/j.envpol.2011.06.008

[14] European Commission. Joint Research Centre. & MSFD Technical Group on Marine Litter. *Guidance on the Monitoring of Marine Litter in European Seas: An Update to Improve the Harmonised Monitoring of Marine Litter under the Marine Strategy Framework Directive*. (Publications Office, LU, 2023).

[15] GESAMP. *Guidelines for the Monitoring and Assessment of Plastic Litter in the Ocean*. *GESAMP - Joint Group of Experts on the Scientific Aspects of Marine Environmental Protection***vol. no 99** (United Nations Environment Programme (UNEP), 2019).

[16] Hanke, G. *et al*. *Title: EU Marine Beach Litter Baselines*. 10.2760/16903 (2019).

[17] de Ramos, B., de Lima, T. M. & da Costa, M. F. Where are Brazil’s marine litter scientific data? *Front. Sustain*. **3** (2022).

[18] Hartmann, N. B. *et al*. Are We Speaking the Same Language? Recommendations for a Definition and Categorization Framework for Plastic Debris. *Environ. Sci. Technol.* **53**, 1039–1047 (2019). 30608663 10.1021/acs.est.8b05297

[19] Wu, D. *et al*. The PlastOPol system for marine litter monitoring by citizen scientists. *Environ. Model. Softw.* **169**, 105784 (2023).

[20] Harley, M. D. & Kinsela, M. A. CoastSnap: A global citizen science program to monitor changing coastlines. *Cont. Shelf Res.* **245**, 104796 (2022).

[21] Medeiros, C., Kjerfve, B., Araujo, M. & Neumann-Leitão, S. The Itamaracá Estuarine Ecosystem, Brazil. in *Coastal Marine Ecosystems of Latin America* (eds. Seeliger, U. & Kjerfve, B.) 71–81. 10.1007/978-3-662-04482-7_6 (Springer Berlin Heidelberg, Berlin, Heidelberg, 2001).

[22] Cheshire, A, Adler, E., Barbière, J. & Cohen, Y. UNEP/IOC Guidelines on survey and monitoring of marine litter. *UNEP Regional Seas Reports and Studies, No. 186; IOC Technical Series* 120 pp. (2009).

[23] Araújo, M. C. B., Santos, P. J. P. & Costa, M. F. Ideal width of transects for monitoring source-related categories of plastics on beaches. *Mar. Pollut. Bull.* **52**, 957–961 (2006). 16797600 10.1016/j.marpolbul.2006.04.008

[24] de Ramos, B., Costa, M. F. & de Lima, T. M. What lies underneath: Comparison among beach litter in the underwater bathing area and exposed beach. *Sci. Total Environ.* **947**, 174661 (2024). 38992372 10.1016/j.scitotenv.2024.174661

[25] de Ramos, B., Costa, M. F. & de Lima, T. M. Marine litter data in Itamaracá Island, Brazil. 2022. 10.6084/m9.figshare.14128610.v1 (2023).

[26] Alkalay, R., Pasternak, G. & Zask, A. Clean-coast index—A new approach for beach cleanliness assessment. *Ocean Coast. Manag.* **50**, 352–362 (2007).

[27] Ramos, B. Photos Marine litter - Itamaracá 2022. *9268883 Bytes figshare*10.6084/M9.FIGSHARE.28695560.V1 (2025).

[28] Ramos, B. *BeachLog data. 31726 Bytes figshare*10.6084/M9.FIGSHARE.27246942 (2025).

[29] Silva, T. *Marine litter _beaches Itamaracá 2022.xlsx. 26301 Bytes figshare*10.6084/M9.FIGSHARE.28939709.V1 (2025).

[30] Ramos, B. Beach Litter, Beach Uses, and Coastal Oceanographic variables in Itamaracá - PE - Brazil. *figshare. Dataset.*10.6084/m9.figshare.29128109.v1 (2025).

[31] Thompson, G. G., Thompson, S. A., Withers, P. C. & Fraser, J. Determining adequate trapping effort and species richness using species accumulation curves for environmental impact assessments. *Austral Ecol.* **32**, 570–580 (2007).

